# Mating Disruption of *Helicoverpa armigera* (Lepidoptera: Noctuidae) Using Yeast-Derived Pheromones in Cotton Fields

**DOI:** 10.3390/insects16050523

**Published:** 2025-05-15

**Authors:** Dimitris Raptopoulos, Petri-Christina Betsi, Neoklis Manikas, Irina Borodina, Maria Konstantopoulou

**Affiliations:** 1 Novagrica Hellas S.A., TESPA “Lefkippos”, 15341 Athens, Greece; dimrapto@novagrica.com; 2 Chemical Ecology and Natural Products Laboratory, Institute of Biosciences and Applications, NCSR “Demokritos”, 15341 Athens, Greece; betsipetri@bio.demokritos.gr (P.-C.B.); neoklismanikas@gmail.com (N.M.); 3 The Novo Nordisk Foundation Center for Biosustainability, Technical University of Denmark, Kemitorvet Building 220, 2800 Kongens Lyngby, Denmark; irina.borodina@fmc.com

**Keywords:** mating disruption, cotton bollworm, sex pheromone, biodegradable, flowable paraffin-based formulation, UAV

## Abstract

The use of insect sex pheromones as an alternative technology for pest control in agriculture and forestry offers a promising solution. The development of a novel technology for the biological production of pheromones through yeast fermentation significantly lowers production costs, enabling the adoption of sustainable pest control practices in field crops, a strategy previously reserved for high-value crops. Over three years of monitoring and mating disruption trials in Greek cotton fields, focusing on the cotton bollworm *Helicoverpa armigera* (Hübner) (Lepidoptera: Noctuidae), it was confirmed that yeast-derived pheromones exhibit equal efficacy compared to their chemically synthesized counterparts. For the mating disruption of *H. armigera*, a biodegradable, flowable, and paraffin-based matrix was developed. The matrix adheres to plants, protects the labile pheromone molecules (*Z*)-11-hexadecenal and (*Z*)-9-hexadecenal, and controls their gradual release into the environment. These biodegradable polymer blobs act as non-retrievable dispensers and can be deployed manually or via unmanned aerial vehicles (UAVs), ensuring efficient and accurate application. This precise, time-efficient, and economically sound technology aligns with European Commission initiatives, such as the Green Deal’s Farm to Fork Strategy and the Biodiversity Strategy, contributing to food sustainability while respecting biodiversity.

## Simple Summary

The increasing need for environmentally sustainable pest management underscores the urgency for alternatives to conventional insecticides. Among these, pheromone-based techniques, particularly mating disruption (MD), are gaining prominence due to their species specificity and environmental safety. However, the widespread use of pheromones is constrained by production costs and limitations in large-scale application methods. This study addresses three primary objectives: (i) to evaluate whether yeast-derived pheromones are as effective as their chemically synthesized counterparts in the MD of *Helicoverpa armigera*; (ii) to validate the suitability of paraffin-based, flowable formulations in retaining and releasing yeast-produced pheromones effectively, and (iii) to assess the feasibility of deploying these formulations over extensive crop areas using UAVs under precision agriculture protocols. Field trials confirmed that yeast-derived pheromones perform equivalently to synthetic ones in both monitoring and disrupting mating of *H. armigera*. Furthermore, the developed paraffin-based, biodegradable formulations demonstrated reliable pheromone release and were compatible with UAV-based deployment. The ability to achieve even distribution over large areas using drones further supports the scalability of this approach. This integrated system, combining biotechnological production, biodegradable formulations, and UAV application, represents a cost-effective, sustainable pest control strategy, offering a practical and eco-conscious solution for modern agriculture that improves food security.

## 1. Introduction

Current trends in managing insect pests of public health and economic significance have increasingly shifted toward biological and biotechnological approaches [1,2,3,4]. This transition is largely driven by the phasing out of many chemical insecticides because of their adverse effects on human health, the environment, and the rise in pest resistance [5]. As a result, there is a growing interest for eco-friendly solutions like pheromone-based strategies that align with the broader goals of sustainability, food safety, and economic feasibility [6]. Unlike broad-spectrum insecticides, pheromones are species-specific, preserve biodiversity, and support ecological balance [7]. Moreover, some pheromones have been shown to exert kairomonal effects on natural enemies, enhancing their efficacy and reinforcing the principles of integrated pest management (IPM) [8].

With over one-fifth of the world’s annual crop production lost to herbivorous insects, there is a compelling need for the production of pheromone-based pest control products in sufficient quantities and at low cost [9]. The agricultural insect pheromone market is projected to reach USD 7.2 billion by 2027 (MarketResearch.com, ReportLinker.com), with mating disruption (MD) solutions becoming more prevalent, particularly in high-value crops, like fruits, vegetables, and nuts. However, pheromone-based pest control is increasingly being extended beyond high-value specialty crops to large-scale field crops, such as cotton, corn, and soybeans [6,10,11]. This expansion is driven by advancements in pheromone production, which address the historically high costs of conventional chemical synthesis of pheromone AIs—making disruption solutions more accessible and economically viable for growers of lower-value crops and commodities [1,12].

Biotechnological production of several insect pheromone components has already been established in yeasts [1,2,13,14,15] and plants [3,4]. Compared to chemical synthesis, biotechnological microbial production offers several important advantages, such as sustainability, reduced environmental impact, and cost-effectiveness [2]. In the case of biotechnological pheromones, biosynthesis byproducts that remain in the final product could potentially influence insect behavior. To address this issue a series of laboratory experiments, including electrophysiological tests (EAG and EAD) and behavioral bioassays, must be performed to confirm the behavioral equivalence of yeast-derived pheromones to chemically synthesized ones. This was observed in *Ostrinia nubilalis*, where the presence of tetradecyl acetate (14:OAc) left in the final product interfered with the insect’s precopulatory behavior [13]. Through biotechnological methods, (*Z*)-11-hexadecen-1-ol and (*Z*)-9-tetradecen-1-ol were produced, among other pheromone compounds, using engineered yeast cell factories. The sex pheromone compounds (*Z*)-11-hexadecenal (*Z*11-16:Ald) and (*Z*)-9-hexadecenal (*Z*9-16:Ald) of the cotton bollworm (CBW) *Helicoverpa armigera* (Lepidoptera: Noctuidae) were produced by oxidation of fermented fatty alcohols to their corresponding aldehydes using tetrakisacetonitrile copper(I) triflate/TEMPO catalyst system. The resulting yeast-derived pheromone was just as efficient and specific for trapping CBW male moths in cotton fields in Greece as the conventionally produced synthetic pheromone mixture [1]. An additional advantage of this biotechnological pheromone production is the capacity to produce both major and minor pheromone components in a single process [1]. This capability is particularly significant, as research has shown that incorporating the minor component *Z*9-16:Ald into sex pheromone formulations enhances both pheromone trap sensitivity and the effectiveness of commercial MD [16].

CBW is the most significant and impactful pest in agriculture in Asia, Europe, Africa, and Australasia, causing damage to crops (cotton, tomato, pigeon pea, chickpea, sorghum, rice, cowpea, and a range of vegetable crops), with an estimated cost of greater than USD 2 billion annually, excluding socio-economic and environmental costs associated with its control [17,18,19]. In addition to feeding on an extensive range of hosts (>180 plant hosts from >45 families), including essential global food and fiber crops, CBW has consistently demonstrated a rapid ability to develop resistance to insecticides, such as organophosphates, pyrethroids, organochlorines and carbamates [20], which has led to pest resurgence in many regions of the world including Greece [21].

Since its identification and the verification of its function [22], the sex pheromone of CBW is widely used for monitoring fluctuations in the pest’s populations. The feasibility of the MD of CBW has been documented since 1996 [23], using slow-release PVC resin formulations (Selibate^®^ HA—AgriSense-BCS Ltd., Pontypridd, UK). However, this method has not gained a hold of the market because of the high cost of chemically synthesized pheromones. Kehat and Dunkelblum [24] conducted MD using Shin-Etsu ropes with satisfactory results in Israel.

Still, in some cases, MD has shown lower control levels compared to conventional insecticides [24,25]. Factors such as field size, crop type, pest population density, local microclimate, and the flight behavior of gravid females can all influence the effectiveness of this technique in the field [26]. In a relevant experiment in tomato, Burgio et al. [26] have concluded that MD can be integrated as a component of IPM to optimize CBW control, either by improving the effectiveness of conventional treatments or by minimizing the frequency of insecticide treatments in areas with high infestation levels.

The aims of this study were threefold, as follows: (a) to verify that yeast-produced pheromones are as effective as chemically synthesized pheromones in controlling CBW populations by MD; (b) to establish that paraffin-based biodegradable formulations can effectively retain, protect, and release the yeast-produced pheromones, like the currently widespread LDPE-based retrievable dispensers (low-density polyethylene), as well as to demonstrate the ease of application via caulking-gun cartridges; and (c) to show that these formulations can be easily and evenly dispersed over large areas using unmanned aerial vehicles (UAVs). The latter is particularly critical for extending the application of MD techniques to crops beyond high-value cash crops, such as in the case of row crops.

## 2. Materials and Methods

### 2.1. Pheromone Formulation

The sex pheromone formulations used for monitoring and the MD of CBW were supplied by Novagrica Hellas SA (Athens, Greece). The monitoring of the CBW adults for both the MD-treated and control plots was carried out using funnel-type traps baited with rubber septa (bromobutyl elastomers) loaded with 2 mg of CBW pheromone, comprising a 97:3 ratio of *Z*11-16:Ald and *Z*9-16:Ald (Bedoukian Research Inc., Danbury, CT 06810, USA).

For the MD, a biodegradable, flowable, and paraffin-based matrix (PheroWax), developed by Novagrica, was utilized to formulate the pheromone compounds. The pheromones used were either chemically synthesized (97:3 ratio, Bedoukian Research Inc., purity > 95%) or a yeast-derived mixture of (*Z*)11-hexadecenal and (*Z*)9-tetradecenal (provided by BioPhero ApS, Copenhagen, Denmark) in 94:6 ratio with a 70% overall purity [8]. Both chemically synthesized and yeast-derived pheromones were integrated into the matrix at approximately 7% AI (*w*/*w*), with their respective technical-grade active ingredients (TGAIs) adjusted to 100% active ingredient (AI). In all field experiments, the pheromone, either yeast-derived or the chemically synthesized active ingredient (AI), was applied at a dosage of 100 g/ha. The final flowable formulations were then packaged in 300 mL caulking-gun cartridges. Emphasis was placed on the protection of the AIs from environmental conditions, via the inclusion of antioxidants and UV protectors.

For aerial application, cartridges and modified dispensing units were securely mounted onto a specially adapted UAV to facilitate dispersal over cotton fields. The UAV used was a custom-built hexacopter designed to carry a payload of up to 10 kg. Standard caulking cartridges, similar to those used in common silicone applicators, were chosen for their versatility. The flight speed was set to approximately 3 m/sec, with PheroWax blobs being dispensed on every other row. Prior to pheromone application, fields were precisely mapped, and the data served to construct a flight plan, which was programmed into the UAV. As a result, the content of the cartridges was evenly dispersed across the cotton fields following the predetermined path based on the GIS data.

### 2.2. Experimental Fields and MD Trials

All field experiments were carried out in the Spercheios Valley in Central Greece, a region where cotton is the predominant cultivation for many consecutive years. Secondary row crops include clover and maize.

#### 2.2.1. First-Year Trial

Initial experiments for the MD of CBW were carried out from July to September 2020. The objectives were as follows: (a) to evaluate the efficacy of yeast-derived pheromone (BIO) compared to chemically synthesized pheromone (CHEM) in disrupting the mating behavior of CBW males and (b) to validate the capability of the biodegradable, paraffin-based matrix to effectively protect the labile CBW pheromone and sustain its male-disorienting effect throughout the cotton growing season until harvest. The experiments were carried out at the following two locations in the Spercheios river valley (Central Greece): plot A (38°53′30.4″ N 22°20′13.7″ E) and plot B (38°52′57.4″ N 22°22′22.3″ E). Each testing plot covered an area of 2 ha, divided into 2 subplots. An additional plot (2 ha), planted with cotton, separated by more than 250 m from the MD-treated plots, received only the grower’s standard practices and served as the control (CO) plot (38°51′22″ N 22°24′17″ E).

In Plot A, a total of 100 g CBW pheromone per ha was applied in the form of PheroWax loaded in 300 mL cartridges. The application was performed manually using caulking guns, forming spherical blobs of approximately 3 g each, which were placed at the base of the cotton branches. The pheromone blobs were distributed (per ha) as follows: (i) CHEM subplot: 50 g of the chemically synthesized CBW pheromone mix containing *Z*11-16:Ald and *Z*9-16:Ald in a 97:3 ratio; (ii) BIO subplot: yeast-derived pheromone equivalent to 50 g CBW pheromone (adjusted to 100% AI). In total, 250 blobs were evenly deposited in each subplot. Each deposited blob contained 200 mg CBW pheromone—either yeast-derived or chemically synthesized, depending on the subplot—adjusted to 100% AI.

In Plot B, a total of 100 g CBW pheromone per ha was applied using low-density polyethylene microtubes (LDPE microtubes, 0.4 mL, Beckman^®^, Brea, CA, USA) divided as follows: 50 g of the chemically synthesized CBW pheromone mix containing *Z*11-16:Ald and *Z*9-16:Ald in a 97:3 ratio in one subplot (CHEM) and 50 g of the yeast-produced pheromone in the second subplot (BIO). The LDPE microtubes were used to obtain a clear comparison between the polymeric matrix and the established LDPE microtube method, allowing for direct evaluations of the degree of protection and the effectiveness of the MD.

The LDPE microtubes were secured by a twist-tie line through their eye-loop directly at the base of the stem of the third leaf from the top of the plant, spaced every 5 plants in alternating planting rows. In total, 250 microtubes were evenly distributed in each subplot. Each microtube was loaded with 200 mg CBW pheromone—either yeast-derived or chemically synthesized accordingly, adjusted to 100% AI. Antioxidants and UV-protectants were incorporated into both types of microtubes.

In all plots, a total of 20 funnel traps (5 in each subplot) were deployed. Each trap was baited with commercially available pheromone dispensers and suspended on wooden poles at a 1.5 m height. Traps were moved 20 m clockwise each week to account for potential pest hotspots in the fields, and males were captured, recorded, and removed once per week. Pheromone dispensers were renewed every four weeks.

This application setup allowed for a direct comparison of the efficacy between the chemically synthesized and yeast-derived pheromone formulations in disrupting the mating behavior of CBW males.

#### 2.2.2. Second-Year Trial

The experiment was repeated in 2021, from July to September, in Spercheios Valley, Central Greece, in two cotton fields of 4 ha each (Plot A: 38°51′37.9″ N, 22°24′24.6″ E and Plot B: 38°52′34.4″ N, 22°23′47.9″ E). Based on the positive results from the previous year, for which the polymeric matrix proved as effective as the LDPE microtubes, we did not include microtubes in the subsequent experiments. Instead, the objective was to scale up the study over a larger area to compare the efficacy of the yeast-derived CBW pheromone (BIO) and synthetic pheromone mix (*Z*11-16:Ald and *Z*9-16:Ald) (CHEM), as well as to explore the feasibility of using the same cartridges for alternative application methods, such as UAVs equipped with PheroWax-releasing devices.

Plot A was divided in two 2 ha subplots (BIO and CHEM), each separated by a 2 ha strip of untreated cotton. In this plot, a modified caulking gun was mounted on a UAV that flew over the subplots in a meander (in zig-zag) pattern, guided by a predetermined flight plan. Every two (2) seconds, the caulking gun was remotely triggered to release a blob of approximately 5 g of pheromone formulation directly onto the cotton plants. After approximately 50 drops, the UAV returned to base to replace the cartridge and then resumed the discharge at the last dropping point. In Plot B (4 ha), the paraffin-based formulation of the yeast-derived pheromone was applied manually using caulking guns. This plot (Plot B) was divided in two subplots (BIO and CHEM), each 2 ha in size. A total of 2000 blobs were evenly deposited on the subplots. Each deposited blob contained 200 mg CBW pheromone, either yeast-derived or chemically synthesized (adjusted to 100% AI). The CO plot was the same one used in the previous year (2020). Four funnel traps were installed in each subplot to monitor the pest population.

#### 2.2.3. Third-Year Trial

In the next year (2022), the experiment was repeated on a much larger scale, covering a total area of 7 ha, using exclusively the yeast-derived pheromone with the same formulation. The pheromone was applied by a UAV equipped with multiple discharging units to optimize the efficiency of the UAV’s flight time. The trial was conducted in the following two plots: a 3 ha plot (38°53′23.2″ N, 22°20′46.1″ E) and a 4 ha plot (38°51′36.30″ N, 22°24′28.97″ E). Prior to the pheromone application, both fields were precisely mapped, and the data were used to generate a flight plan, which was then programmed into the UAV. Consequently, the contents of the cartridges were evenly distributed across the cotton fields, following the predetermined path based on the GIS data. Four funnel traps were installed in each plot to monitor the pest population. The pheromone formulation dispersed in the form of uniform blobs of 5–6 g, and the polymer was evenly applied at a rate of 100 g AI/ha.

Over the three consecutive years, 4 funnel traps were installed in the CO plot; trap catches were collected weekly and served the same function as all of the others in the MD-treated plots.

### 2.3. Assessment of MD Efficacy

The efficacy of the MD was assessed by (i) calculating the percentage of male inhibition according to the formula {[CO − MD)/CO] × 100}, representing the average male catches in the untreated and treated plots, through weakly collection from pheromone-baited traps; (ii) evaluating the pest larval population present in the MD and CO plots through visual inspections of the cotton plants; and (iii) recording the cotton yields in the control and pheromone-treated plots provided by the farmers.

Damage was assessed through visual inspections of the cotton plants in both the MD and CO plots, conducted four times during the experimental period (approximately every three weeks). During each inspection, a random zig-zag pattern was followed, and twenty plants per subplot were examined. The leaves, combs, and other fruiting structures of the upper four nodes of the plant were examined for signs of damage or presence of larval. The damage was calculated as the ratio of the cotton damage to the total samples.

### 2.4. Determination of the Amounts of Pheromone Released from the Polymer Blobs

#### 2.4.1. Calibration Curve

To determine the amount of Z11-16:Ald released from the polymer blobs at various time points, a Carboxen/polydimethylsiloxane fiber (CAR/PDMS, 75 μm, Supelco, Bellefonte, PA, USA) was used for the solid-phase microextraction (SPME) analysis. Stock solutions of Z11-16Ald were prepared in pentane. Aliquots of 50, 100, 200, 400, 800, and 1200 ng were placed in 50 mL headspace glass vials, sealed with PTFE septum screw caps, and covered with parafilm. All calibration samples were prepared in triplicate. Next, the SPME fiber was exposed in the headspace above the sample for 45 min at 55 °C and was finally inserted into the GC injector (GC Agilent 7890B, column HP-5). The column temperature program was held at 50 °C for 1 min and then programmed up to 250 °C at 5 °C/min. The initial temperature of the on-column injector was set at 50 °C for 1 min and then programmed to rise to 220 °C at 180 °C/min. The detector temperature was set at 250 °C. Helium was the carrier gas. The amounts of pheromone were calculated from the calibration curve using standard solutions of Z11-16:Ald (R^2^ = 0.99). The analyses were performed in triplicate.

#### 2.4.2. Pheromone Release Rate

Τo determine the amount of pheromone released from the polymer blobs at various time points, a Carboxen/polydimethylsiloxane fiber (CAR/PDMS, 75 μm, Supelco) was used for the SPME analysis, as described by Kikionis et al. [27]. Spherical samples of the formulated pheromone were placed on 5 × 3 cm paper cards. The average weights of the CHEM pheromone mix and BIO were 2.18 ± 0.02 and 2.15 ± 0.02 g, respectively. All samples were naturally aged under field conditions near the experimental plots, from July to September for the three consecutive years, with temperatures ranging from 24 to 42 °C. Three samples of each treatment were collected every week for 8 weeks and placed in 50 mL headspace glass vials, sealed with PTFE septum screw caps, covered with parafilm, and stored at −20 °C until analysis. The analyses were conducted by GC under the same conditions as described above. The amount of *Z*11-16Ald released from the pheromone formulations was calculated from the aforementioned calibration curve using standard solutions.

### 2.5. Data Analysis

The field data were subjected to analysis of variance (ANOVA) (SAS Institute, Cary, NC, USA, 2000) to study the differences observed between the MD and control plots (*p* < 0.05), as well as to detect significant differences between the treatments. The data are presented as the mean of male catches per trap per week. All statistical analyses were performed using SPSS v. 22 (IBM, Chicago, IL, USA).

## 3. Results

**Figure 1:**
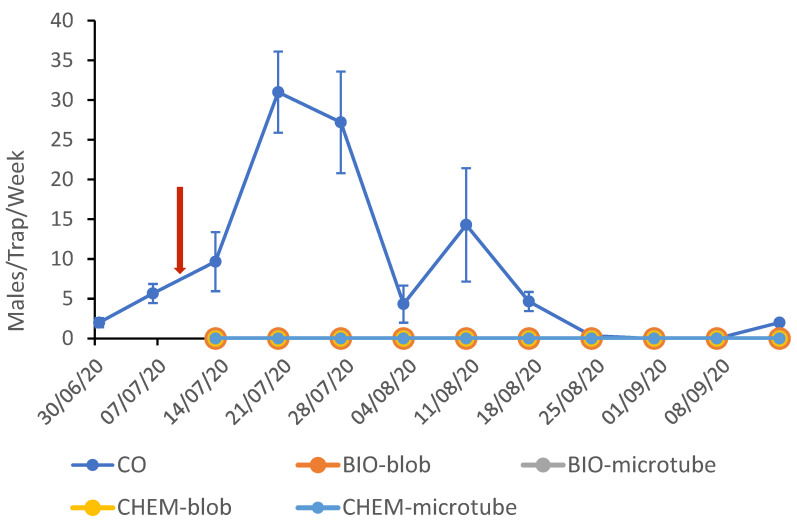
Weekly CBW trap catches (±SE) in the plots after mating disruption application in 2020. The yeast-derived and the chemically synthesized pheromones were dispensed from PheroWax (BIO-blob and CHEM-blob) and microtubes (BIO-microtube and CHEM-microtube). The arrow marks the date of the mating disruption application.

The mean weakly catches per pheromone trap for the CO and MD plots are shown in Figure 1 for the 2020 trials. After the pheromone application on July 10th, a complete silencing of the traps in the MD plots was observed, since the trap catches were zero during the flight period until harvest. The two generations of CBW were well distinguished, even though the pest population was not very high, as recorded in the CO plot. The peak of the 2nd generation was on July 20th (CO: 31.0 ± 5.1, BIO-blob: 0.0, and BIO-microtube: 0.0) and for the 3rd on August 10th (CO: 14.3 ± 7.1, BIO: 0.0, BIO-blob: 0.0, and BIO-microtube: 0.0).

The trap shutdown for CBW was effectively achieved in the treated plots. The yeast-derived pheromone, whether formulated in PheroWax or in microtubes, was equally as efficient as the chemically synthesized on, prepared in the same manner (F = 47.053, d = 23, *p* = 0.000). The sexual disorientation of the pest lasted throughout the flight period and across the two consecutive generations of the pest.

In 2021, high levels of CBW populations were recorded in the area where the field trials were conducted. Despite this, a complete silencing of the traps was observed in the pheromone-treated plots (both with chemically synthesized and yeast-derived pheromones) (F = 42.041, d = 19, *p* = 0.000).

**Figure 2:**
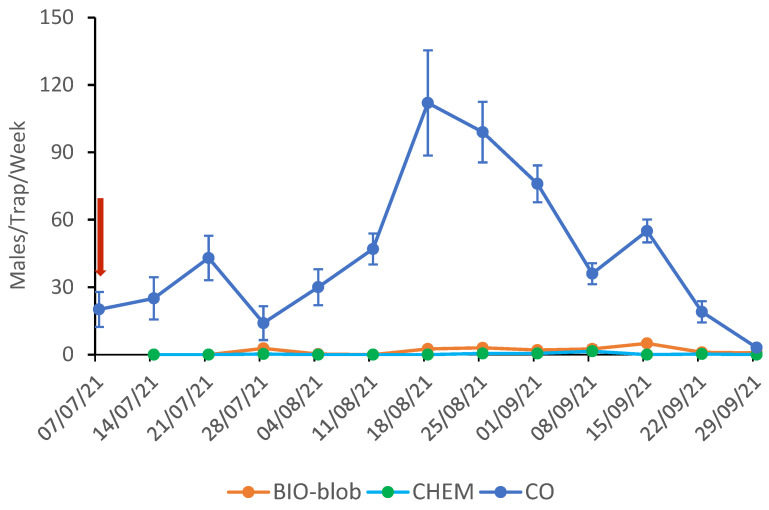
Weekly CBW trap catches (±SE) in the plots after the mating disruption application in 2021. The yeast-derived (BIO-blob) and the chemically synthesized (CHEM) pheromones were formulated in PheroWax. The arrow marks the date of the mating disruption application.

The mean weakly catches per pheromone trap for the CO and MD plots are shown in Figure 2 for the 2021 trials. Notably, following the pheromone application on July 7th, the traps in the MD plots showed zero male captures throughout the monitoring period. The two generations of CBW were well distinguished, but the 2nd population density was lower than the 3rd, maybe due to high temperatures. The peak of the 2nd generation was on July 21st (CO: 43.0 ± 9.9, BIO: 0.0, and CHEM: 0.0) and for the 3rd on August 18th (CO: 112 ± 23.4, BIO: 2.5 ± 0.3, and CHEM: 0.0).

**Figure 3:**
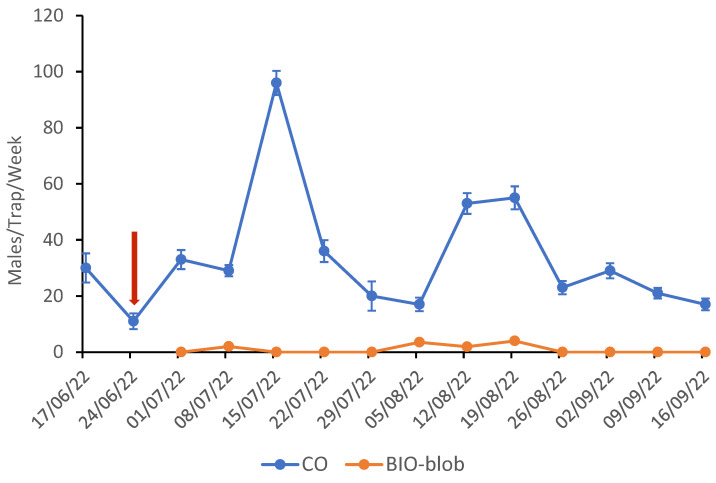
Weekly CBW trap catches (±SE) for the plots after the mating disruption application in 2022. The yeast-derived pheromone was formulated in PheroWax (BIO-blob). The arrow marks the date of the mating disruption application.

The mean weakly catches per pheromone trap for the CO and MD plots are shown in Figure 3 for the 2022 trials. After the pheromone application on June 24th, a complete silencing of the traps in the MD plots was observed. The two generations of CBW were well distinguished. The peak of the 2nd generation was on July 15th (CO: 96 ± 4.3 and BIO-blob: 0.0) and for the 3rd on August 19th (CO: 55 ± 4.1 and BIO-blob: 4.0 ± 0.4). For the 3rd year of the trials, the mating disorientation of the males was successfully achieved with the use of the yeast-derived pheromone compared with the control plots (F = 51.024, d = 15, *p* = 0.000).

**Figure 4:**
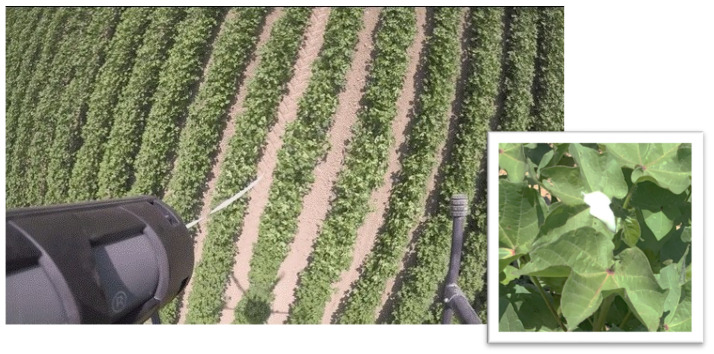
PheroWax blob dropping from the cartridge nozzle (**left**). PheroWax blob deposited on the cotton plants (**right**).

With the UAV application, instead of being sprayed, the blobs were precisely dropped onto the crops, guided by GIS technology (Figure 4).

**Figure 5:**
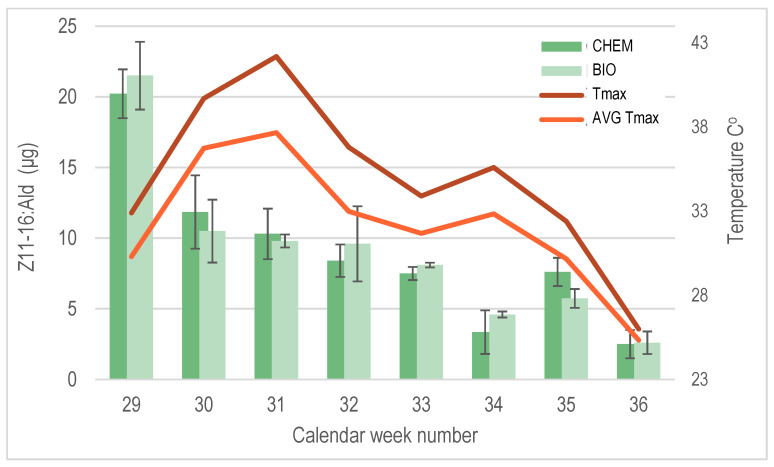
Z11-16:Ald release rate from the chemically synthesized (CHEM) and yeast-derived pheromones (BIO), both formulated in PheroWax for the 2021 trial.

The determination of the release rate of the major pheromone compound Z11-16:Ald from PheroWax confirmed that the yeast-derived and chemically synthesized pheromones exhibited similar performances. Both formulations effectively maintained the required pheromone levels in the field, ensuring sustained CBW sexual disorientation throughout the entire growing season (Figure 5).

The PheroWax matrix provided robust protection for the aldehydes, ensuring their stability even during the elevated temperatures experienced during the summer of 2021. *Z*11-16:Ald remained detectable in the field for up to nine weeks after application, maintaining sufficient levels to effectively sustain CBW sexual disorientation.

After three years of trials, the results from monitoring the traps confirm that communication disruption was fully achieved. In particular, the calculated suppression ratios of the total male captures were 100%, 99.2%, and 99.4% in 2020, 2021, and 2022, respectively.

Lower damage to the cotton buds, squares, and bolls was observed in the MD-treated plots over the three consecutive years compared with the control plots. In the MD-treated plots, the damage was notably lower in all years (2020: 2.1%; 2021: 3.8%; and 2022: 3.3%) than in the CO plots (2020: 3.1%; 2021: 4.2%; and 2022: 4.4%).

The crop yield data confirm the effectiveness of the MD application, demonstrating successful pest control without the use of chemical insecticides. For 2020, the cotton yield data between the MD-treated and control fields were comparable, with yields of 2850 kg/ha and 2900 kg/ha, respectively. In 2021, the yields were recorded as 2960 kg/ha for MD-Bio, 2900 kg/ha for MD-Chem, and 3000 kg/ha for the control. In 2022, the MD-treated fields outperformed the control, yielding 4000 kg/ha compared with 3700 kg/ha, respectively.

## 4. Discussion

With ongoing biotechnological advancements in pheromone synthesis and formulation, pheromone-based strategies are becoming increasingly economically viable for pest management, including MD [28], even for low-value crops, such as rice, soybean, cotton, and maize. This shift supports the broader adoption of sustainable agricultural practices. MD is widely recognized for its high efficacy, environmental compatibility, and user safety, aligning with the principles of the European Commission Regulation (EC) No. 1107/2009. Moreover, while chemical insecticides face a growing number of resistance cases, there are currently only a few documented instances of resistance to MD using pheromones, highlighting its robustness as a pest management strategy [7,29].

Our results demonstrate that the yeast-derived pheromone (*Z*11-16:Ald and *Z*9-16:Ald in a 94:6 ratio), applied at a rate of 100 g AI/ha, effectively disrupted the orientation of the CBW males toward female pheromone sources, just like chemically synthesized pheromones. Even during 2021 and 2022, when the CBW populations were notably high and temperatures in the cotton fields were elevated, successful trap shutdown was achieved, and the crop yield remained high. The larger-scale MD trials conducted in 2022 to evaluate the sustainability of this method for CBW population management, also yielded positive results [30].

The flowable, biodegradable pheromone formulation tested demonstrated long-lasting effectiveness, maintaining sufficient pheromone concentrations in the field throughout the entire flight period (over eight weeks) despite extremely high temperatures. The matrix adheres well to plants, providing protection to the labile pheromone molecules (*Z*11-16:Ald and *Z*9-16:Ald) while ensuring their controlled, gradual release into the environment. Biodegradable dispensers enhance the sustainability of pheromone-based technologies by eliminating plastic waste at the end of the season, thus avoiding the need for disposal in landfills. Landfills store up to 42% of the worldwide plastic waste and serve as an important source of microplastics (MPs), which are considered emerging pollutants [31]. Flowable formulations function as “matrix-type” dispensers, where the active ingredient is uniformly distributed within a polymer matrix. These dispensers can be manufactured with varying viscosities and are easily applied at ambient temperature using various methods. Once applied, they consistently release the active ingredient. Their formulation resists being washed away by rainfall or irrigation and, over time, they erode and decompose naturally in the soil. Based on the outcomes observed over three years of applying yeast-produced pheromones formulated in a flowable, biodegradable matrix, it has been conclusively demonstrated that CBW can be effectively managed using the tested active ingredients and the associated methodologies, whether through manual or UAV applications.

A significant advantage of the biopolymer flowable matrices forming the biodegradable blobs is that, from a regulatory standpoint, they are classified as single-point, non-retrievable, and biodegradable dispensers that do not require retrieval at the end of the season. They are not sprayed and can be applied either manually or via UAV. When deployed via UAVs, the blobs are accurately dropped onto the crop using GIS-guided systems and precision agriculture techniques. This aspect of our MD technology is particularly important, as it is well-established in the literature [26] that the pattern of the dispenser’s distribution over a larger area, extending beyond the cultivated field, plays a crucial role in the effectiveness of the technique. Kerns [32] and de Souza et al. [33] also highlighted this, emphasizing that the application of the dispensers should not be confined to the field but also extend to adjacent areas.

UAV technology allows for the implementation of precision agriculture practices over a wide area. Drones are particularly well-suited for medium to large fields, especially in areas close to inhabited regions where the use of sprays, light aircraft, and helicopters is not permitted. Drone-assisted mating disruption (MD) offers the following key advantages: (i) precision through GIS-guided dispenser placement; (ii) faster application compared to manual methods; (iii) reduced labor costs; and (iv) reliable deployment even under challenging weather conditions. These benefits make drones a highly effective and efficient tool for pheromone-based pest management [34,35,36]. To the best of our knowledge, this is the first reference regarding the successful use of yeast-derived pheromones for pest control through MD. Wang et al. [3] demonstrated the disruption of mating of CBW in bean fields using plant-derived pheromones, specifically from genetically modified *Camelina sativa* seeds. Our approach represents a novel application by utilizing yeast-derived pheromones, which are cost-effective and sustainable for large-scale pest management.

Our MD trial, which used trap shutdown as an indicator of the suppression of male orientation to mates, shows that the yeast-derived pheromone formulations were as effective as high-purity synthetic pheromone formulations at disrupting mating. The yeast-derived pheromones successfully interfered with the males’ ability to locate sex pheromone sources in the field, a fundamental behavioral mechanism behind MD. This finding suggests that yeast-derived pheromones could play an important role in establishing commercial pest population suppression via MD. Similar success with MD using different types of dispensers has been reported for various crops, including cotton in Israel [24], lettuce in Japan [37], cotton in Pakistan [23], and tomatoes in Italy [26]. This reinforces the potential for yeast-produced pheromones to be effectively applied in pest control strategies. The results from these field trials suggest that yeast-derived pheromone could effectively replace synthetic chemical pheromones in pest management products. This shift would enable pheromone-based pest control products to compete with conventional pesticides, thereby reducing global reliance on environmentally hazardous chemicals. The trials will be replicated in larger fields also using lower doses of pheromone per treated area.

Like chemically synthesized pheromones, bio-based pheromones offer several benefits, including effectiveness against pests resistant to traditional pesticides, support for integrated pest management and organic farming, and the ability to produce agricultural products free from insecticide residues. They also enhance crop protection because of their high specificity, safeguard non-target species, and prevent secondary pest outbreaks. Moreover, they are derived from renewable feedstocks.

We successfully formulated yeast-derived pheromones in plant protection products, primarily in the form of flowable biodegradable matrices that can be delivered by UAVs. This system is optimized for medium to large farmlands, including both arable crops and orchards, while implementing precision agriculture practices. This precise, time-efficient, and economically viable technology aligns with European Commission initiatives, such as the Green Deal’s Farm to Fork Strategy and the Biodiversity Strategy, promoting food sustainability and respecting biodiversity. Notable applications of this technology include maize, cotton, soybeans, and field tomatoes, among others.

Conclusively, new, environmentally friendly, and low-carbon-footprint pheromone production methods, combined with advanced formulation systems, offer efficient pest control without leaving behind persistent organic pollutants (POPs). These innovative production methods, when delivered to crops using equally cutting-edge technologies for their application, offer a sustainable efficient and cost-effective solution for pest control, promoting eco-friendly agricultural practices.

These innovative production methods, when delivered to crops using equally cutting-edge technologies, such as drones for their application, offer a sustainable, efficient, and cost-effective solution for pest control, promoting eco-friendly agricultural practices.

## Data Availability

The original contributions presented in this study are included in the article. Further inquiries can be directed to the corresponding author.
